# Building a Pipeline to Increase Academic Workforce Diversity to Achieve Health Equity

**DOI:** 10.1089/heq.2020.0091

**Published:** 2021-03-22

**Authors:** Girardin Jean-Louis

**Affiliations:** ^1^ Department of Population Health, NYU Grossman School of Medicine, New York, New York, USA.; ^2^ Department of Psychiatry, NYU Grossman School of Medicine, New York, New York, USA.

**Keywords:** workforce diversity, mentoring, training, health equity

## Abstract

The disproportionately low number of under-represented minority (URM) faculty pursuing research careers is attributed partly to an inadequate pool of well-trained URM scientists. This is compounded by lower rates of successful competition for NIH funding by URM scientists. Evidence shows black scientists are 13% less likely to receive NIH funding relative to white scientists. Increasing the number of well-trained URM scientists is a highly significant goal, achievable through exposure to mentored learning opportunities in an autonomy-supportive academic network. In this article, the author describes his academic career trajectory leading to the establishment of the NHLBI-funded PRIDE Institute. The institute's overarching goal is to increase the number of URM scientists pursuing academic careers to address important cardiovascular health disparity issues. The PRIDE institute has been very successful in achieving 2020 Healthy People goals of a greater academic workforce diversity.

## Introduction

Growing up, I heard repeatedly that I should become an educator. I ignored those injunctions because I believed my aptitude and penchant best suited me for a career in engineering. For most, adolescence is a time to forge one's path; however, for me that was a tall order given both my father and mother were educators. My dad was a high school principal and my mother, a middle-school teacher.

In 1986, I immigrated to the United States from Haiti intent on pursuing an engineering degree. A family friend suggested I attend City College of New York and so I did. In the second year of college, my school advisor recommended I explore other disciplines for a well-rounded education. After a quick glance through the college's course offerings, he suggested I join a sleep laboratory. The mere suggestion piqued my curiosity, and after my advisor told me I would receive a stipend, the deal was sealed. Then, I was unfamiliar with the National Institutes of Health, but I liked the idea of being a paid learner. This was my first NIH fellowship award, which marked the beginning of my career in sleep and circadian sciences. To my parents' surprise, their son was no longer going to be an engineer; rather, one who was destined to become a leading sleep research scientist and educator. Needless to say, they thought I had lost my mind. If you are familiar with the Caribbean culture, you may know what I mean.

The transition from engineering to sleep and circadian sciences did not come without its challenges. However, where there are challenges, opportunities to learn and grow exist. My college mentor had to juggle the responsibilities of training students in recruiting study participants, conduct electroencephalography recordings, curate data, and write scientific articles because of a lack of NIH funding. This presented many challenges, but I took it as an opportunity to learn how to run a research laboratory on a low budget. The research had to be conducted, and in those days, we did not have Google Scholar, PDFs, or the internet. We had to go to the library, make photocopies, and scroll through microfiche, which was not always convenient. The research process was not always convenient; however, I enjoyed the idea of learning about something I truly liked.

Recognizing my dedication to sleep and circadian research, my late mentor, Dr. Art Spielman, convinced me to enroll in a doctoral program. He believed, if I wanted to have an impact on the field, I needed a Ph.D. I agreed with the understanding that much of my training would require me to go above and beyond what is typically required of doctoral students. To succeed, I had to train research assistants and labor tenaciously to generate the data for a Ph.D. Moreover, I had to build a research laboratory with limited laboratory resources. My experience was quite unique given most graduate students enter doctoral programs with already established laboratories. However, this was necessary as I intended to keep my promise to finish in 4 years. The goal to complete a Ph.D. in 4 years was an anomaly, as my classmates often reminded me. It took me a while to realize why they believed it took so long to complete a Ph.D.

I now understand they were alluding to the structural hurdles that are difficult for under-represented minority (URM) students to overcome. Such hurdles are often compounded by the imposter syndrome and stereotype threat. Perhaps my naiveté, engendered in part by my Caribbean worldview, made it possible to overlook these barriers. I reasoned these were obstacles all students had to overcome to succeed. Recent events have highlighted that structural racism is seemingly an insurmountable hurdle URM students have to face in academia, which often discourage them from achieving their career goals. Indeed, evidence suggests that URM scholars are less likely to receive NIH funding, relative to their white counterparts.^1^

Despite the skepticism and lack of support from faculty in the doctoral program, I did build the laboratory. They did not realize how determined I was. With the support of my mother, a devout Christian, and her church community, they prayed for my success. Dismayed by my strength and determination, some faculty were perplexed by my accomplishments. One said, “That's a bad example.” “That's anathema!” Another said. “You cannot have graduate students build their own lab.” Well, I did, and because of this daring move, I completed my Ph.D., as planned. Building the laboratory was no small feat, as it necessitated long hours of work and dedication as well the contribution of friends with technical skills, bound by the common purpose to achieve something special. Yes, “it takes a village.” This was also made possible by a bank loan to purchase electronic devices to shore up the data collection apparatus and cover operational costs. At the time, I was told that this was not a wise decision; one that would not have any return on investment. I took the risk, and I dare say: it paid dividend.

Later, I made an observation that left an indelible mark on my mind. For 3 years, all the students I trained were white. This observation became even more troubling when, to my great consternation, I noted I was the only black presenter attending my first annual sleep conference in 1992. “This was unacceptable,” I said to myself. This had to change if I were to attend the meeting annually. Every year thereafter, I brought several URM students to the meeting. I cannot take credit for a diversified sleep academy, but I know I contributed to it.

Upon completion of my postdoctoral training with Dr. Dan Kripke at the University of California, San Diego, in 2001, I returned to New York where I built three more laboratories. This was only possible because of my previous experience, although each one presented with its unique challenges. As you might imagine, given the lack of NIH funding at the time, the common denominator in all of these initiatives was that the workforce to support research needed training. I did, and many of the students I trained now occupy high-level positions in academia or are in private practice, mostly serving vulnerable communities plagued by the burden of cardiovascular disease.

In 2005, a major shift in my approach to training occurred after meeting Dr. Luther Clarke, who had recently become the director of a new NIH-designated Health Disparities Center at SUNY Downstate, tasked to train URM physician-scientists. I joined the team and established a robust training and mentoring infrastructure. A year later, the center received its first R25 award (Program to Increase Diversity among Individuals Engaged in Health-Related Research-PRIDE).^2–5^ Given Dr. Clark's enormous responsibility as chief of cardiology, he entrusted me to run the program.

We now had funding to implement a well-regimented program. However, since the program was new, it was a struggle to enroll the first cohort of URM faculty. Some could not devote two consecutive weeks to participate given their clinical responsibilities; others had to teach. The first year would have been a complete failure had we not been able to convince three scholars to enroll. In the proposal, however, we said we would easily enroll 12 scholars a year. Upon realizing we had fallen short of our goal, Dr. Jared Jobe, the PO, was gracious and accommodating, encouraging us to do better next year. Well, we did. We had five. The year after, we enrolled 8, and since then we have been enrolling 12 yearly.

Well, the success was so compelling that NIH added six new sites. At NYU, the home of the Behavioral Medicine and Sleep Disorders Research PRIDE Institute, we have trained 150 URM scholars, leveraging the PRIDE Model to build other training programs: one focusing on stroke disparities, another on neuroscience research, and yet another on aging and brain health. The PRIDE Model is also being implemented globally, including a program in Ghana and another in Jamaica. Most of these physician-scientists are currently in the frontline, conducting innovative research to optimize delivery of personalized care through telemedicine and caring for individuals in vulnerable communities severely affected by coronavirus disease 2019 (COVID-19).

As of 2019, the NIH-funded PRIDE sites trained 600 URM faculty, excelling in all indicators of academic success. We found significant increases in the number of scholars promoted to higher academic ranks. The number of publications increased, and so have the number of NIH awards. What's more, our last comparative analysis revealed PRIDE Scholars were twice as likely as their counterparts to receive NIH funding to support their research.^2^ Perhaps, the impact of the PRIDE Institute is best captured in the following two quotes from graduating scholars: “As far as I know, nothing else provides the type of mentorship I needed. As a URM and working with people that understand some of the unique challenges that come along with that and help you to apply things in that light [it] doesn't exist in most places.” “The issue with PRIDE is that the mentors are someone like you, who went through what you went through; so, they understand what you are going through. If they can make it, I can make it also. In a lot of minority training programs, the PI is non-minority and probably can't relate to what you are going through.”

## Conclusions

PRIDE has done well, but there are miles to go before it reaches its full potential.^2^ In the next few years, we expect that it will encompass the full spectrum of training opportunities at all academic levels, starting at the high school through postdoctoral levels. This would achieve the national mandate to build a sustainable pipeline to increase the academic workforce diversity.

Programs such as the PRIDE Institute could not be more needed at this time; particularly when URM faculty are questioning structural racism, impeding their academic success. As a program director, I have engaged in listening sessions with our scholars for the past few months where scholars were asking existential questions: Does my research matter? Will it count toward promotion or tenure? I said to them: “The PRIDE Mentorship Team is determined to support you to ensure your academic success.” “It will not rest until all the objectives of your Individual Development Plan will have been met.”

My parents could not be prouder of me for having followed my passion while helping others achieve their dream to build successful academic careers, focusing on the national mandate to achieve health equity for all U.S. communities.

## Abbreviations Used

COVID-19: coronavirus disease 2019
URM: under-represented minority
